# Glycometabolism Reprogramming of Glial Cells in Central Nervous System: Novel Target for Neuropathic Pain

**DOI:** 10.3389/fimmu.2022.861290

**Published:** 2022-05-20

**Authors:** Erliang Kong, Yongchang Li, Mengqiu Deng, Tong Hua, Mei Yang, Jian Li, Xudong Feng, Hongbin Yuan

**Affiliations:** ^1^ Department of Anesthesiology, Changzheng Hospital, Second Affiliated Hospital of Naval Medical University, Shanghai, China; ^2^ Department of Anesthesiology, The No. 988 Hospital of Joint Logistic Support Force of Chinese People’s Liberation Army, Zhengzhou, China

**Keywords:** inflammation, glycolysis, microglia, astrocyte, synapse, neuropathic pain, glycometabolism reprogramming

## Abstract

Neuropathic pain is characterized by hyperalgesia and allodynia. Inflammatory response is conducive to tissue recovery upon nerve injury, but persistent and exaggerated inflammation is detrimental and participates in neuropathic pain. Synaptic transmission in the nociceptive pathway, and particularly the balance between facilitation and inhibition, could be affected by inflammation, which in turn is regulated by glial cells. Importantly, glycometabolism exerts a vital role in the inflammatory process. Glycometabolism reprogramming of inflammatory cells in neuropathic pain is characterized by impaired oxidative phosphorylation in mitochondria and enhanced glycolysis. These changes induce phenotypic transition of inflammatory cells to promote neural inflammation and oxidative stress in peripheral and central nervous system. Accumulation of lactate in synaptic microenvironment also contributes to synaptic remodeling and central sensitization. Previous studies mainly focused on the glycometabolism reprogramming in peripheral inflammatory cells such as macrophage or lymphocyte, little attention was paid to the regulation effects of glycometabolism reprogramming on the inflammatory responses in glial cells. This review summarizes the evidences for glycometabolism reprogramming in peripheral inflammatory cells, and presents a small quantity of present studies on glycometabolism in glial cells, expecting to promote the exploration in glycometabolism in glial cells of neuropathic pain.

## Introduction

Neuropathic pain is caused by a lesion/disease of the somatosensory system, and is estimated to affect 7%-10% of the general population (1, 2). The pathogenesis of neuropathic pain is complex, and involves the entire nociceptive pathway (primary afferent nerves, spinal cord, brain, and descending pathways) as well as glial cells.

The pathological basis of neuropathic pain is hyperalgesia and allodynia caused by synaptic remodeling in the nociceptive pathway. Chronic nerve injury promotes the release of pro-inflammatory cytokines to activate intracellular signal transduction pathways, and to disturb the balance between facilitation and inhibition in pain signal transduction. A variety of animal models for neuropathic pain (e.g., chronic constriction injury and spinal nerve ligation) have been developed based on persistent nerve injury (3). Recent studies identified abnormal glycometabolism in neurons and the supporting glial cells upon chronic nerve injury (4). Under normal oxygen-rich conditions, pyruvate enters the tricarboxylic acid (TCA) cycle for oxidative phosphorylation into CO_2_ and NADH in the central nervous system. Under hypoxic conditions, however, pyruvate is converted into lactate and NAD^+^ through anaerobic glycolysis. Anaerobic glycolysis has low efficiency in energy production than oxidative phosphorylation, but is the preferred metabolic pathway in the active phase of cell proliferation (5).

Glial cells are implicated in a variety of neurophysiological processes, including neuronal development, synaptic remodeling, and neuropathic pain (6). Glial cells have been shown to participate in chronic pain *via* multiple mechanisms, including regulating glutamate concentration in synaptic cleft through glutamate transporters (7), controlling the release of neurotransmitters (8), altering stability of the synaptic microenvironment (9), and modifying inter-neuronal communications (10). When activated by inflammation, glial cells switch from oxidative phosphorylation to preferentially use glycolysis as energy source, with accompanying changes in pentose phosphate pathway, amino hexanoic acid and glutamine hydrolysis pathway (11). Metabolic intermediates produced in glycometabolism reprogramming also provide substrates for other biosynthetic pathway in cell growth and differentiation, and could regulate a variety of intracellular signaling pathways at both the transcriptional and post-transcriptional levels. To some extent, these effects determine the fate of neurons or glial cells (12).

This review summarizes the neuropathology of neuropathic pain, regulation of phenotypic transition and pain sensitization by glycometabolism reprogramming of glial cells under chronic nerve injury, and the influences of glycometabolism reprogramming on synaptic plasticity and neuronal excitability. The viewpoints are helpful in exploring the crucial roles of glycometabolism reprogramming of glial cells in the development of neuropathic pain and providing potential targets for the intervention of neuropathic pain.

## Neuropathology of Neuropathic Pain

Nociceptive stimuli are converted into electrochemical signals by pain receptors and transmitted to the spinal cord *via* primary afferent neurons. As the station of signal regulation and integration, the spinal cord sends pain signals to the brain through the upward projection fibers. The upward signal transmission is regulated by downward signals through the spinal cord to effectors *via* efferent neurons (13). Changes in any part of this nociceptive pathway can lead to allodynia or hyperalgesia.

**Figure 1:**
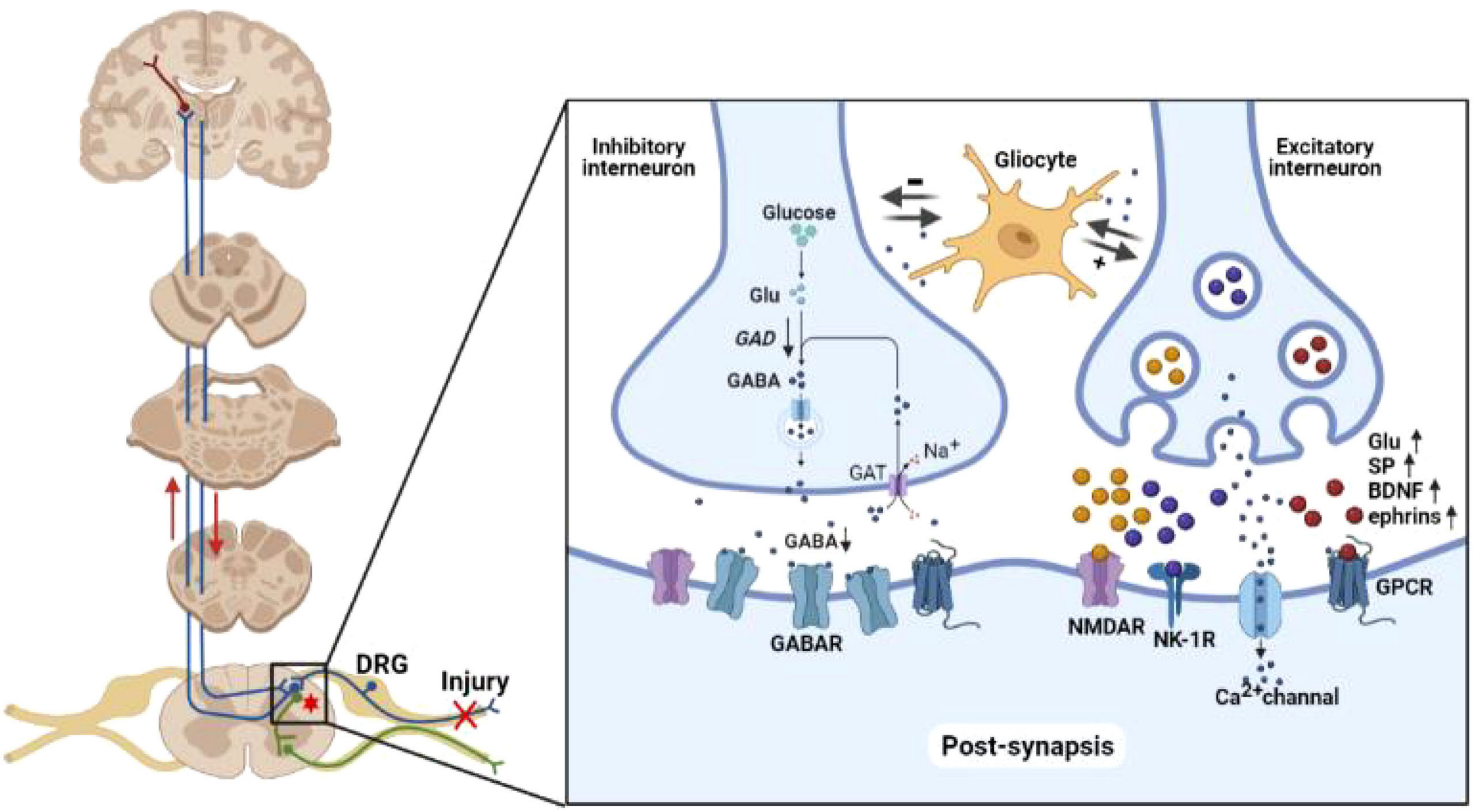
Neuropathology of neuropathic pain. Peripheral nociceptive stimuli are converted into electrochemical signals by pain receptors and transmitted to the brain through the spinal cord *via* upward projection fibers. The spinal cord also relays downward signal to regulate the nociceptive signal transmission. The imbalance of inhibitory/excitatory interneurons forms the basis of neuropathic pain. In inhibitory interneurons, GABA is synthesized from glutamate by GAD and released into the synaptic cleft. GABA in the synaptic cleft is taken up by interneurons *via* GABA transporters in a Na^+^-dependent mechanism. Chronic nerve injury reduces the activity of GABA synthase and GAD, ultimately resulting in disinhibition. Chronic nerve injury enhances the synthesis of glutamate, SP, BDNF and ephrins in excitatory interneurons. These neurotransmitters enhance the spontaneous excitatory postsynaptic currents mediated by AMPAR, NMDAR, NK-1R, and Ca^2+^ channel on postsynaptic membrane. Glu, glutamate; GAD, glutamic acid decarboxylase; GABA, γ-aminobutyric acid; GAT, GABA transporter; SP, substance P; BDNF, brain derived neurotrophic factor; NMDAR, N-methyl-D-aspartate receptor; NK-1R, neurokinin 1 receptor; GPCR, G protein-coupled receptor.

Chronic nerve injury could produce structural changes in the spinal projection area of afferent neurons. In physiological conditions, peripheral C fibers mainly project to the substantia gelatinosa (lamina II) of spinal cord to transmit chronic pain signals, whereas the Aδ fibers mainly project to lamina I and III to transmit acute pain signals. Tactile information is mainly transmitted by Aβ fibers that project to lamina III and IV. In neuropathic pain, chronic nerve injury induces abnormal projection of the Aβ fibers to neurons in lamina I and III to form additional neural circuits for hyperalgesia and allodynia. Chronic nerve injury also induces actin cytoskeleton remodeling, thus changing the density and length of dendritic spines of the neurons in spinal cord (14, 15). Rho/Rac molecules in the GTPase superfamily could transmit pain signals to intracellular actin cytoskeleton through neurotransmitters, and promote the generation of dendritic spines and ultimately structural communication among neurons. Selective inhibition of Rac1 protein has been shown to attenuate hyperalgesia and reduce the changes in dendritic spines in an animal model of neuropathic pain (16). Chronic nerve injury also promotes autophagy and apoptosis in the inhibitory γ-aminobutyric acid (GABA) interneurons, and reduces the activity of GABA synthase and glutamic acid decarboxylase (GAD), ultimately leading to neuronal disinhibition (17). As the supporting cells of neurons, glial cells are exquisitely sensitive to microenvironment changes. Activated glial cells secrete a variety of substances to promote interneuron sensitization. Intrathecal injection of a microglia activation inhibitor has been shown to attenuate hyperalgesia in a neuropathic pain model by reducing the expression of inflammatory factors in spinal microenvironment (18). Chronic nerve injury also affects a variety of other signaling molecules in central nervous system, including growth factors, neurotransmitters, intracellular second messengers, nuclear transcription factors and membrane receptors. Central sensitization seems to be the result of complex interaction among these mechanisms and disturbed balance between excitatory and inhibitory synapses (19) (**Figure 1**).

Synaptic plasticity is implicated in the development and ageing of the central nervous system, as well as the pathophysiology of a number of diseases, including Alzheimer’s disease and neuropathic pain. Synaptic plasticity can be categorized into functional (changes in information transmission) or structural (changes in information storage). Examples of functional synaptic plasticity included long-term potentiation (LTP; strengthening of synaptic connection) and long-term depression (LTD; weakening of synaptic connection). LTP is typically occurs in large synapses and dendritic spines whereas LTD tends to occur in small synapses (20). Upon repeated input of nociceptive signals from peripheral nerves to the spinal neurons, excitatory postsynaptic currents (EPSC) mediated by α-amino-3-hydroxy-5-methyl-4-isoxazolepropionic acid (AMPA) on postsynaptic membrane tend to increase over time. This process requires synergistic activation of the N-methyl-D-aspartate (NMDA) receptor, neurokinin 1 (NK1) receptor and low-threshold T-type Ca^2+^ channel (21). Chronic pain is mainly transmitted to the interneurons in lamina II of spinal cord through C fibers. Chronic depolarizing stimulation removes the conformational block of voltage-dependent Mg^2+^ channel inside the NMDA receptor of the postsynaptic membrane, and enhance the sensitivity of neurons to subsequent stimulation. Chronic stimulation of C fibers also promotes the platform currents of L-type Ca^2+^ channel on spinal neurons to further enhance neuronal excitability (22). This complex process involves both excitatory and inhibitory transmitters, as well as neuromodulators such as substance P (SP) and brain derived neurotrophic factor (BDNF), G protein-coupled receptors (GPCR), NK-1 and tyrosine kinase B (TrkB) (23) (**Figure 1**).

## Glycometabolism Reprogrammin in Glial Cells

Glucose is the main source of energy in central nervous system. Glucose enters neurons mainly actively through glucose transporter (Glut) on cell membrane (24). Pyruvate, the final product of glycolytic process, enters the TCA cycle in mitochondria under normal oxygen-rich conditions but is converted to lactate under hypoxic conditions. Pyruvate generates 32 ATP molecules through the TCA cycle and electron transport chain in mitochondria and only 2 ATP molecules upon conversion to lactate by lactate dehydrogenase (LDH) (25). Pyruvate dehydrogenase (PDH) is a rate-limiting enzyme in the TCA cycle, and is regulated (inhibited) *via* phosphorylation by pyruvate dehydrogenase kinase (PDK). When PDH is phosphorylated, pyruvate is shunted from the TCA cycle to anaerobic glycolysis (26). Four human PDK subtypes have been identified. PDK_1_ is activated in anoxic environment; PDK_2_ is activated upon acetyl-CoA and NADH accumulation; PDK_3_ is active in high-ATP environment; PDK_4_ plays a vital role upon starvation. Increased PDK_2_ and PDK_4_ expression has been found in spinal cord neurons in a diabetic model for neuralgia, and double knockout of PDK_2_ and PDK_4_ could alleviate hyperalgesia *via* inhibiting synaptic accumulation of pro-inflammatory factors and lactate and subsequent changes of ion channel permeability in neurons as well as glial cells (27). In primary culture of spinal neurons, exogenous lactate increases the permeability of cell membrane and alters the electrophysiological properties of synapses by facilitating calcium influx through calcium ion channels. The PDK inhibitor dichloroacetate and LDH inhibitor FX11 partially alleviate hyperalgesia in diabetic neuralgia model, providing a potential target for treatment of diabetic neuralgia (28).

### Chronic Nerve Injury Promotes Microglia Activation by Enhancing Glycolysis

Microglia and astrocytes play critical roles in neuroinflammation (29). Microglia are resident immune cells in the central nervous system, and could activate inflammasome, NF-κB and other inflammatory signaling pathways; in contrast, astrocytes are mainly involved in regulating the integrity and permeability of blood-brain barrier (30). In the resting state, microglia primarily rely on oxidative phosphorylation of glucose as energy source. Upon activation, microglia shift from the TCA cycle to anaerobic glycolysis (31) *via* multiple mechanisms, including increased expression of pro-inflammatory factors and accumulation of advanced glycation end products (AGEs) (32). Microglia activation also promotes autophagy and apoptosis of neurons. Short-term exposure to β amyloid shifts microglia from oxidative phosphorylation to glycolysis *via* the mammalian target of rapamycin/hypoxia inducible factor-1α (mTOR/HIF-1α) pathway (33). Long-term exposure to β amyloid, however, attenuated both glycolysis and oxidative phosphorylation, and reduced the responsiveness of microglia to noxious stimuli. In a mouse model for Alzheimer’s disease, exogenous interferon-γ (IFN-γ) attenuated neurological deficits by attenuating the stimulation of β amyloid to microglia through promoting glycolysis by activating mTOR pathway (33). In further studies, mice with TREM-2 knockout in Alzheimer’s disease showed decreased mTOR pathway activity, impaired glycolysis and increased neuronal autophagy (34). Enhanced glycolysis in activated microglia has also been noted in patients with multiple sclerosis, an autoimmune disease (35), and has been explored as potential target in the treatment of multiple sclerosis (36).

**Figure 2:**
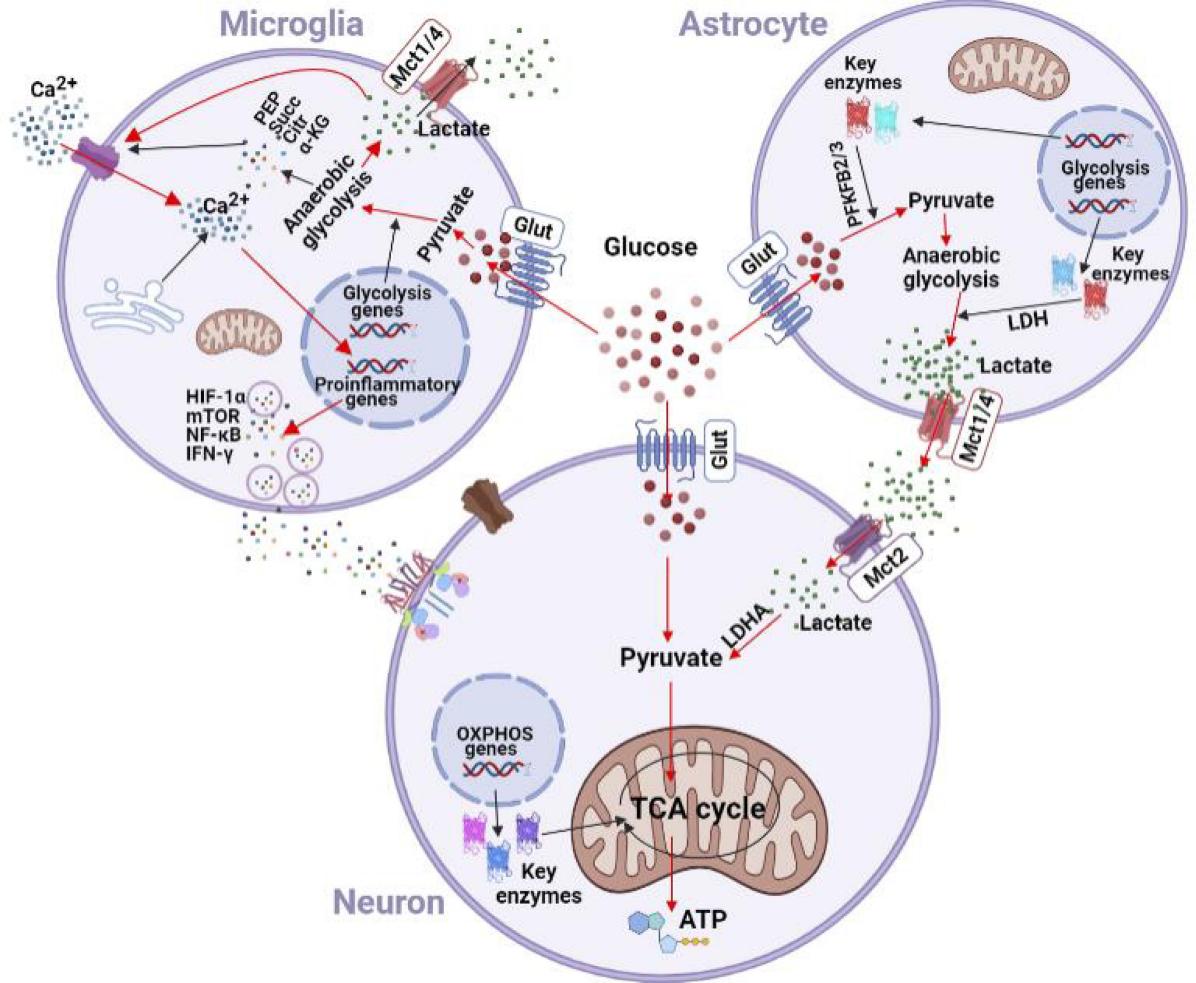
Effects of chronic nerve injury on glycometabolism in microglia and astrocytes. Glycolysis is enhanced in microglia under hypoxic and inflammatory insults. Enhanced glycolysis leads to accumulation of lactate, PEP, succinate, citric acid, and α-KG. Lactate is transferred into synpatic microenvironment by the lactate shuttle to facilitate the Ca^2+^ channel in cytomembrane and inhibit the Ca^2+^ channel in the endoplasmic reticulum. The resulting increase of Ca^2+^ concentration in cytoplasm activates inflammatory pathways (e.g., NF-κB, HIF-1α, mTOR, and IFN-γ) to promote the transcription of pro-inflammatory factors in microglia. Astrocytes express high levels of PFKFB_2/3_, and could metabolize glucose into pyruvate. Upon chronic nerve injury, glutamate is taken up by astrocytes in a Na^+^-dependent mechanism, which in turn increases intracellular Na^+^ concentration and activates Na^+^-K^+^-ATPase on the cell membrane to promote glucose uptake and induce anaerobic glycolysis. Lactate is then transferred out of astrocytes *via* Mct1/4, and enters neurons *via* Mct2. Glut, glucose transporter; Mct, monocarboxylate transporter; PEP, phosphoenolpyruvic acid; Succ, succinate; Citr, citric acid; α-KG, α-ketoglutarate; HIF-1α, hypoxia inducible factor-1α; mTOR, mammalian target of rapamycin; IFN-γ, interferon-γ; PFKFB, 2, 6-phosphofructo-2-kinase; LDH, lactate dehydrogenase; LDHA, lactate dehydrogenase A; TCA, tricarboxylic acid; OXPHOS, oxidative phosphorylation.

In addition to increased glycolysis at the expense of TAC cycle, glucometabolic reprogramming in microglia also features enhanced glutamine hydrolysis and pentose phosphate pathway. All together, these metabolic changes lead to the accumulation of a variety of intermediates, including phosphoenolpyruvic acid (PEP), succinate, citric acid, methylene succinic acid, α-ketoglutaric acid, lactate and 2-hydroxyglutaric acid, which in turns alters the acid-base balance in microenvironment, promotes transcription of pro-inflammatory factors and activates inflammatory signaling pathways to change the inflammatory phenotypes of both microglia and peripheral immune cells (37, 38). Upon activation of T cells by chronic nerve injury, PEP accumulation interferes with Ca^2+^ signaling and promotes the inflammatory cascade (39). When T cell receptor (TCR) is activated by antigen, cell membrane permeability increases and Ca^2+^ enters the cytoplasm to activate a number of signaling pathways. PEP accumulation by glycolysis inhibits Ca^2+^ channels in endoplasmic reticulum, thus preventing Ca^2+^ from entering into the Ca^2+^ reservoir in endoplasmic reticulum. These changes increase Ca^2+^ concentration in cytoplasm, further activating inflammatory pathways to maintain the activated state of peripheral immune cells and promoting the transcription of pro-inflammatory factors. PEP accumulation produces similar effects in macrophages, including the induction of M1 polarization and increased expression of pro-inflammatory factors (40). The responses of resident microglia to the glycolysis metabolites are also characterized by a shift towards the inflammatory phenotype (41) (**Figure 2**).

### Chronic Nerve Injury Promotes Lactate Transfer Between Astrocytes and Neurons

Astrocytes are also part of the resident immune system in the central nervous system, and could release cytokines or chemokines upon activation. Similar to glial cells, astrocytes also undergo glycometabolism reprogramming during the development of neuropathic pain (42). A1 astrocytes are characterized by activation of the classical complement cascade, which in turn promotes neuropathic pain by disrupting the stability and function of synaptic structures. In contrast, A2 astrocytes are characterized by upregulation of neurotrophic factors, which in turn play protective roles during the process of neurodegeneration (43).

Recent *in vivo* and *vitro* studies have demonstrated that the astrocyte-neuron lactate shuttle (ANLS) is a crucial additional source of energy for neurons, especially under stress and continuous neuronal stimulation (44, 45). Lactate produced in astrocytes can be transported out of cells and enters neurons by monocarboxylate transporters (MCTs). MCTs of different subtypes have cell-specific distribution. MCT_1_ and MCT_4_ have low affinity for lactate and are mainly distributed on glial cells to mediate the outward transport of lactate, whereas the high-affinity MCT_2_ is mainly distributed on neurons and mediate the uptake of lactate. In comparison to the very low glycogen storage capacity in neurons, astrocytes have high glycogen storage. Upon sensing enhanced energy requirements by surrounding neurons, astrocytes increase lactate production through glycolysis to provide metabolic substrates for neurons (46, 47). In comparison to neurons, astrocytes also express higher levels of 2,6-phosphofructo-2-kinase 2 (PFKFB_2_) and PFKFB_3_, and could thus provide energy to neurons *via* activated glycolysis (44). Accumulated glutamate in synapses upon chronic nerve injury enters astrocytes *via* glutamate transporters (GLT), increases intracellular Na^+^ concentration and activates Na^+^-K^+^-ATPase on cell membrane of astrocytes. This in turn promotes glucose uptake and induces glycolysis. Anaerobic glycolysis can also produce ATP, which is used for *de novo* glutamate synthesis, glutamate transfer between astrocytes and neurons, and the maintenance of Na^+^-K^+^-ATPase function (48). Dependence of neurons on lactate of astrocyte origin varies among different species, and disruption of glycolysis in astrocytes has been shown to result in loss of neurons in drosophila (49). Metabolic dependence of neurons on lactate of astrocyte origin, and relevance to neuropathic pain are illustrated in **Figure 2**.

### Lactate Participates in the Synaptic Connections Between Neurons

Under normal conditions, lactate concentration is 10 to 50 times higher than pyruvate, and can be released into blood to provide energy for tissues and organs except in the central nervous system. Neurons only use glucose from the blood for energy metabolism in physiological condition, whereas glial cells could provide additional energy source for neurons under stress. Both *in vitro* and *in vivo* studies have shown that lactate concentration gradient from astrocytes to neurons plays an important role in neuronal stability, apoptosis and energy metabolism (25, 50). The lactate concentration gradient from astrocytes to neurons could be seen with two-photon microscopy (51). Lactate in astrocytes upregulates learning-related genes and produce intercellular connectivity changes in interneurons (52). Exposure to lipopolysaccharide or interferon increases lactate shuttle between microglia and neurons, indicating that microglia could also provide lactate for neurons under stress (53). Glutamine and glutamate that enter into astrocytes from synaptic cleft could enter the TCA cycle. Since the blood-brain barrier is impervious to lactate, astrocyte-neuron lactate shuttle is critical for neuronal survival, memory and synaptic remodeling under hypoxic conditions or inflammation (54, 55) (**Figure 2**).

## Glycolysis and Pain Sensitization

Glycometabolism reprogramming is critical in pain sensitization. Reciprocally, transformation of glycometabolism is subject to epigenetic regulation by intermediates and co-factors, thus forming a feedback regulation system between glycometabolism and pain sensitization (56). Glycometabolism usually affects the expression of pain related genes by regulating substrates required for gene modification. By disrupting the NAD^+^/NADH balance, glycometabolism transformation can alter the function of histone deacetylase since the catalytic process of acetylase requires a certain concentration of NAD^+^. Histone deacetylase has been implicated in pain signal transmission and regulation (57). For example, lactate may have opposite roles in different stages of the inflammatory process (58). In the early stage of macrophages activation in peripheral nervous system, lactate accumulation is generated by glycolysis conversion to promote histone acetylation, which in turn upregulate anti-inflammatory genes. In a sense, lactate serves as a feedback signal that switches macrophage to anti-inflammatory phenotype in the development of inflammation. Lactate dehydrogenase A (LDHA), an enzyme that catalyzes the conversion between pyruvate and lactate, regulates the expression of IFN-γ in T cells *via* acetyl-CoA. In CD4^+^ T cells, acetyl-CoA is mainly used for histone acetylation of IFN-γ promoters, thereby promoting T cell differentiation into Th1 subsets (59). Reduction of acetyl-CoA levels by ATP-citrate lyase (ACLY) knockout reduces the expression of key enzymes in glycolysis (e.g., hexokinase 2, PFKFB and LDHA), whereas exogenous lactate reduces the effects of ACLY knockout on glycolysis (60). Under low glucose conditions, exogenous lactate enters CD8^+^ T cells and is converted into acetyl-CoA to increase IFN-γ expression (61). Succinate has been shown to inhibit the DNA methylase TET family genes in Treg cell subsets to alter cell proliferation (62). Changes in the intermediates in glycolysis in macrophages, T cells and other immune cells have been implicated in the abnormal excitability in primary sensory nerve fibers and dorsal root ganglion during pain sensitization and synaptic plasticity. Parallel glycometabolism reprogramming has also been found in spinal cord, where the nociceptive signal is integrated (63).

### Effects of Lactate on Pain Sensitization

It is well known that lactate participates in various physiological processes as a signal molecule, and it may exhibit anti-inflammatory effects in some states. Lactate inhibits toll-like receptor induction of inflammasome and production of IL-1β *via* the GPR81-mediated suppression of innate immunity (64, 65). In dendritic cells, lactate accumulation drives the transformation of inflammatory phenotype by regulating the secretion of interleukin-10 (IL-10) (66). Lactate has been shown to inhibit the migration and cytotoxicity of CD8^+^ T cells and promote the proliferation of Treg cells *via* its action on the key enzymes in glycolysis (67). In certain conditions, however, lactate accumulation could enhance the immune response and inflammatory cascade mediated by the NF-κB pathway in Th17 cells (68). Similarly, in endothelial cells, lactate accumulation stimulates the NF-κB/IL-8 pathway and induces the production of reactive oxygen species (ROS), resulting in increased cytomembrane permeability and blood-brain barrier disruption (69). Lactate has also been implicated in ROS production in myogenic cells under stress conditions and could up-regulate the expression of multiple genes related to oxidative stress and pro-inflammatory activities (70). The inflammatory effects and oxidative stress induced by lactate in periphery contribute to pain sensitization. In the brain and spinal cord, lactate accumulated under chronic nerve injury can enter neurons and microglia and serve as a substrate in the TCA cycle to generate both ATP and ROS. Immune regulatory factors and ROS cascades produced by microglia promote apoptosis and autophagy, change the stability and permeability of ion channels, and contribute to pain sensitization by depolarizing synaptic membrane and remodeling (31, 71).

### Effects of Succinate on Pain Sensitization

Succinate could be accumulated upon TCA cycle disruption and enhanced glutamine decomposition. Accumulation of succinate in synovial macrophages in response to lipopolysaccharide (LPS) exposure directly inhibits M1 polarization caused by proline hydrolase, thereby promoting stable expression of HIF-1α and IL-1β (72), and inhibiting the transcription of anti-inflammatory cytokine IL-10 (73). Extracellular succinate binds to succinate receptor 1 (SUCNR1) to modulate pro-inflammatory/anti-inflammatory signal pathways in nociceptive neurons. Knockout of the SUCNR1 gene in myeloid cells in adipose tissue has been shown to promote the expression of pro-inflammatory genes in macrophages, adding support to the anti-inflammatory function of succinate (74). Extracellular succinate could produce pro-inflammatory action under different conditions. For example, extracellular succinate has been shown to promote the production of prostaglandin E2 in neural stem cells to exert pro-inflammatory effect (75). Succinate can affect macrophage migration and increase the secretion of pro-inflammatory cytokines, including tumor necrosis factor α (TNF-α) and IL-1β, in dendritic cells (76). Succinate also indirectly enhances IFN-γ and TNF-α production in effector T cells during antigen presentation (77).

### Effects of Citric Acid on Pain Sensitization

Citric acid is generated during the TCA cycle in mitochondria and transported to the cytoplasm by the citrate carrier (CIC) to participate in the synthesis of fatty acids. In the cytoplasm, citric acid is metabolized by ACLY to acetyl-CoA and oxaloacetate. This process also promotes ROS and nitric oxide (NO) synthesis. CIC knockout reduces citric acid level in the cytoplasm and the production of ROS, NO and prostaglandins to prevent transition to the pro-inflammatory phenotype (78). Citric acid is converted into cis-aconitate in mitochondria and transferred into cytoplasm for oxidative dealkylation to methylene succinic acid. Methylene succinic acid accumulates in large quantities in M1 macrophages and acquires anti-bacterial properties when the TCA cycle is disturbed (79). Disruption of methylene succinic acid synthesis using gene knockout techniques has been shown to induce the expression of pro-inflammatory factors (80). In general, methylene succinic acid exerts its anti-inflammatory effects primarily *via* two signal transduction pathways: one to directly inhibit succinate dehydrogenase to downregulate pro-inflammatory factors, and the other to activate the transcription factors, including nuclear factor E2-related factor 2 (Nrf2) and activating transcription factor 3 (ATF3), in macrophages (81). Through these mechanisms, citric acid participate in synaptic remodeling in pain sensitization.

### Effects of Other Metabolic Intermediates on Pain Sensitization

Several other metabolic intermediates in glycolysis also contribute to pain sensitization. In peripheral monocytes, β-glucan increases cytoplasmic fumaric acid levels and reduce the activity of KDM5 demethylase and promotes cell migration (82). α-Ketoglutarate (α-KG) accumulation induces gene expression related to M2 polarization in macrophages through histone demethylation, promotes the transformation of macrophages to M2 phenotype and enhances their anti-inflammatory activity (83). α-KG seems to produce opposite action in T cells. The IL-2 signal pathway can increase α-KG accumulation in T cells to induce their differentiation into the Th1 subset. α-KG also increases the expression of pro-inflammatory and glycolysis genes through DNA methylation modification, which in turn promotes the glycolysis process in T cells (84). 2-HG, a structural analogue of α-KG, competitively inhibits α-KG-dependent histone demethylase and promotes histone hypermethylation. Increased production of 2-HG under hypoxic conditions increases the hypermethylation of histone and DNA by inhibiting URX and TET2 proteins to increase the expression of CD62L and CD127 and promote T cell differentiation into memory cells to induce synaptic plasticity and pain sensitization (85). Abnormal accumulation of S-adenosylmethionine (SAM) also alters the synaptic microenvironment. LPS stimulation of macrophages promotes histone trimethylation by increasing SAM production and SAM/S-adenosine homocysteine ratio, resulting in the upregulation of pro-inflammatory genes. *In vivo* and *in vitro* studies have confirmed that reduced production of SAM caused by methionine deficiency could alter the inflammatory state of primary afferent neurons and dorsal root ganglion neurons by inhibiting T cell proliferation and cytokine production (86). In summary, accumulation of metabolic intermediates in glycolysis could alter the phenotype of immune and glial cells through different pathways to participate in the development of pain sensitization and synaptic plasticity.

## Effects of Glycometabolism Reprogramming on Synaptic Plasticity

### Microglia and Synaptic Plasticity

Microglia serve as sensors to detect changes in the microenvironment in the central nervous system (87). Through modifying synaptic pruning, microglia regulate experience-dependent plasticity in the barrel cortex and visual cortex after removal of monocular deprivation. Microglia in the resting state plays a vital role learning and memory. Upon infection, injury or stress, microglia migrate to the site of inflammation, assume an amoeboid shape and secrete cytokines, chemokines and ROS (88). Metabolic changes in neurons and glial cells upon injury or stress (e.g., lactate, succinate and citric acid) are important in the formation of synaptic plasticity in pain sensitization and neuropathic pain, as well as a variety of other central nervous system diseases (89).

Microglia are exquisitely sensitive to acidic metabolites. Exposure of microglia to exogenous lactate increases the mRNA of thioredoxin interacting protein (TXNIP) to accelerate neuronal apoptosis and autophagy. This process causes the irreversible changes in synaptic structure and function in the progression of chronic pain, vascular dementia and Alzheimer’s disease (90). H^+^ could act as a second messenger to regulate the activity of voltage-gated Ca^2+^ channels, NMDA and GABA receptors. Blocking NMDA receptors could alter the expression of genes related to lactate-induced LTP and LTD, suggesting NMDA receptors are key downstream signal molecules of lactate (91). In addition, lactate and ROS can also promote neuro-inflammation by activating NMDA receptors to mediate Ca^2+^ influx and the downstream signal cascade. The Src family kinases (SFKs) phosphorylate NMDA receptor subunits to promotes Zn^2+^ entry into cells through NMDA receptor channels to participate in the activation of the TrKBs/ERK pathway, and ultimately synaptic remodeling and learning/memory (92, 93). A negative correlation between pH and lactate levels in the synaptic microenvironment has been found in patients with schizophrenia or bipolar disorder (94). Overall, the effects of glycometabolism reprogramming in microglia on pH homeostasis in the synaptic microenvironment are important to synaptic plasticity and development of neuropathic pain.

**Figure 3:**
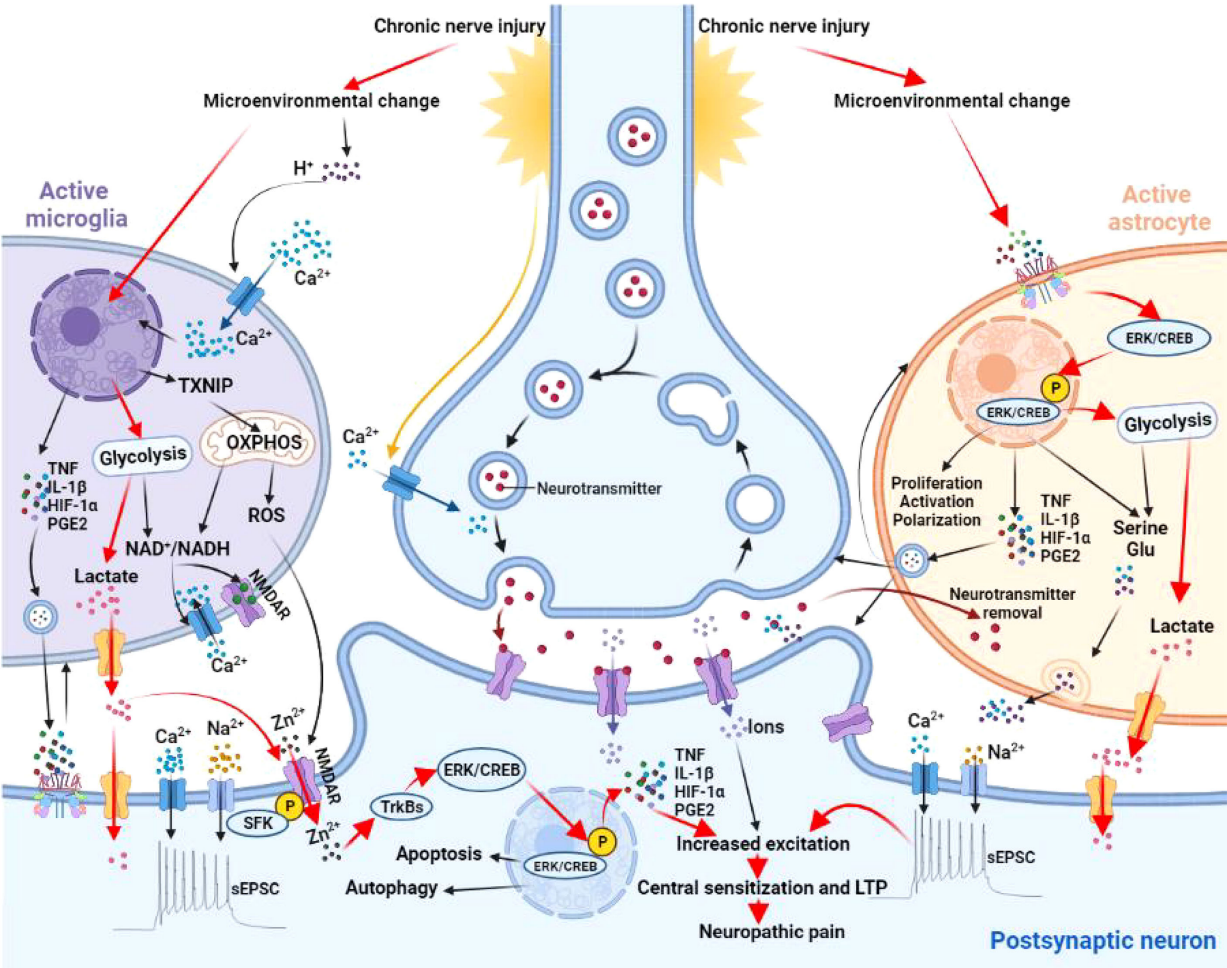
Effects of microglia and astrocyte activation on synaptic plasticity. Microglia sense changes in synaptic microenvironment and H^+^ accumulation activates a series of stress responses to shift microglia into activated state. These changes promote the transcription and expression of TXNIP, key enzymes of glycolysis and proinflammatory mediators (e.g.,TNF, IL-1β, HIF-1α, and PGE2). TXNIP is closely related to oxidative stress in producing ROS to increase cytomembrane permeability. Increased expression of key enzymes in glycolysis promotes lactate production and disrupts the balance of NAD^+^/NADH. An imbalanced NAD^+^/NADH ratio activates the NMDA receptor and increases Ca^2+^ influx to trigger downstream inflammatory cascade. Increased concentration of proinflammatory mediators in the vicinity of synapses promotes synaptic plasticity and polarization of microglia itself. Activation of NMDA receptors, Ca^2+^ influx and the downstream signaling cascade are regulated by SFKs and TrKB receptors. SFKs phosphorylates NMDA receptor subunits to promotes Zn^2+^ entry into postsynaptic neurons through NMDA receptor channels to activate the TrKBs/ERK pathway. Phosphorylation of TrKBs/ERK promotes autophagy, apoptosis, and neuroinflammation. Astrocytes also sense the changes in synaptic microenvironment and activates the ERK/CREB signaling. Upon entering the nucleus, *p*-ERK activates p-CREB to initiate related gene transcription and promotes proliferation, activation, and polarization. Phosphorylation of ERK/CREB also enhances glycolysis and proinflammatory mediators, leading to the accumulation of lactate, glutamate, and serine. All together, these changes increase the excitability of postsynaptic neurons and contribute to central sensitization in neuropathic pain. TXNIP, thioredoxin interacting protein; OXPHOS, oxidative phosphorylation; ROS, reactive oxygen species; NMDAR, N-methyl-D-aspartate receptor; HIF-1α, hypoxia inducible factor-1α; TNF, tumor necrosis factor; IL-1β, interleukin-1β; PGE2, prostaglandin E2; SFK, Src family kinases; ERK, extracellular signal regulated kinase; CREB, cyclic AMP response element binding protein; LTP, long-term potentiation; Glu, glutamate; sEPSC, spontaneous excitatory postsynaptic current.

Changes in the NAD^+^/NADH ratio caused by glycometabolism reprogramming in microglia alter the redox status in synaptic microenvironment. Lactate accumulation increases NADH in both neurons and microglia, and upregulates the expression of a variety of genes, including BDNF, Arc and Zif268 to influence synaptic plasticity (95). Reduced NAD^+^/NADH ratio activate NMDA receptors and increases Ca^2+^ influx to trigger downstream inflammatory cascade. Imbalanced NAD^+^/NADH ratio also affects the levels of transcription factors, deacetylase activity and Ca^2+^ pathways in microglia (96, 97). The Ca^2+^ pathway is a critical link in the lactate signal process in glycometabolism reprogramming. Many other key pathways in synaptic plasticity, including the ERK/CREB pathway, dopamine D2 receptors, metabotropic glutamate receptors (mGluRs) and cannabinoid receptors, are regulated by Ca^2+^ (98). NAD^+^-dependent sirtuin-1 signal pathway also participate in synaptic plasticity by regulating the expression of plasticity-related genes (e.g., BDNF, Arc and Zif268) and modifying dendritic morphology of neurons. To sum up, cellular redox state and particularly NAD^+^/NADH ratio are key steps between glycometabolism reprogramming and synaptic plasticity (99) (**Figure 3**).

### Astrocytes and Synaptic Plasticity

Recent studies indicated that lactate produced through glycogenolysis in astrocytes is critical for long-term memory formation. Disturbing the lactate shuttling between astrocytes and neurons impairs memory formation and consolidation, whereas exogenous lactate attenuates such effects. Astrocyte-neuron lactate shuttle in the spinal cord participates in the formation of abnormal neural circuits in persistent hyperalgesia in neuropathic pain (46). Repeated exposure to noxious stimuli result in substantial changes in astrocyte. Chronic peripheral nerve injury has been shown to promote the proliferation of astrocytes in the spinal cord, as evidenced by increased expression of astrocyte marker glial fibrillary acidic protein (GFAP). Activated astrocytes release a variety of inflammatory mediators and metabolites (e.g., TNF-α, IL-1β, ATP, glutamic acid and serine) to enhance spontaneous excitatory postsynaptic currents and reduce inhibitory interneuron activities, ultimately leading to central sensitization in neuropathic pain (100, 101). Chronic nerve injury has also been shown to increase glutamate concentration in the synaptic cleft *via* inhibition of glutamate transporters (7).

Synaptic sensitization largely depends on the activation of neuronal receptors and ion channels, intracellular signal transduction pathways and related gene expression. Central sensitization in neuropathic pain shares some common molecular mechanisms with LTP in hippocampus (102). For example, lactate transfer from astrocyte to neuron and subsequent NMDA receptor activation and downstream intracellular signal pathways could alter the synaptic plasticity in context of synaptic plasticity in the spinal cord as well as in LTP in the hippocampus in mice. Disrupting the astrocyte-neuron lactate shuttle has been shown to results in amnesia by impairing LTP in the hippocampus (103).

In the central nervous system, glycogen is stored primarily in astrocytes as a reservoir of energy source upon glucose deprivation. In contrast, glycogen store is practically absent in neurons. In comparison to adult brain, neurons in developing brain (rats less than 3 weeks of age) expresses less glucose transporters but more MCTs, indicating the crucial role of lactate in energy metabolism during development of the central nervous system (104). Intraventricular administration of selective inhibitors of MCT or phosphoenolpyruvate carboxykinase (a key enzyme in lactate metabolism) attenuates the proliferation and differentiation of neuronal precursor cells in newborn mice (105). Lactate in the general circulation can also affect synaptic function. Increased lactate in blood by strenuous exercise has been implicated in synaptic remodeling by stimulating the synthesis of vascular endothelial growth factor (VEGF) *via* hydroxy-carboxylic acid receptors on vascular endothelial cells (106, 107).

Plantar injection of complete Freund’s adjuvant (CFA) increases lactate release in the anterior cingulate cortex; inhibiting glycolysis in anterior cingulate cortex, in contrast, alleviates CFA-induced chronic inflammatory pain (108). Knockout of MCT genes in neurons of the anterior cingulate cortex also alleviates CFA-induced inflammatory pain, indicating a prominent role of lactate transfer from astrocytes to neurons in central sensitization and neuropathic pain. Numerous studies have demonstrated a critical role of phosphorylated ERK and activation of the transcription factor cyclic AMP response element binding protein (CREB) in synaptic plasticity and central sensitization. Inhibition of glycolysis has been shown to block the ERK/CREB pathway. Specifically, exogenous lactate may participate in synaptic remodeling and central sensitization in neuropathic pain by enhancing the phosphorylation of ERK and CREB (109, 110). Overall, compromised energy supply to neurons due to disruption of the lactate shuttle between astrocytes and neurons contribute significantly to synaptic plasticity and neuropathic pain *via* multiple mechanisms (111) (**Figure 3**).

## Conclusion

Increasing evidence suggests important role of glycometabolism reprogramming in microglia and astrocytes in neuropathic pain. Glycometabolism reprogramming in microglia promotes the transformation of microglia to the pro-inflammatory phenotype and increases ROS production. The resulting changes in synaptic microenvironment are important pathological basis for pain sensitization. Astrocytes provide energy support to surrounding neurons *via* astrocyte-neuron lactate shuttle. In addition to a substrate for TAC cycle, lactate that enters the neurons from astrocytes also serves as a signal molecule to promote synaptic plasticity by regulating a variety of signaling pathways. In summary, a plethora of information showed that glycometabolism reprogramming of glial cells contribute to hyperalgesia and allodynia in neuropathic pain and represent potential targets for developing novel treatment for neuropathic pain.

## Author Contributions

EK, YL, and MD drafted the manuscript; TH, MY, JL, and XF designed and prepared the figures; HY conceived the study. All authors made significant contribution and approved the submitted version.

## Funding

This work was supported by National Natural Science Foundation of China (81971046 and 82171220) and Medical Science and Technology Research Program of Henan Province (SBGJ202003056, SBGJ202102204 and LHGJ20200781).

## Conflict of Interest

The authors declare that the research was conducted in the absence of any commercial or financial relationships that could be construed as a potential conflict of interest.

## Publisher’s Note

All claims expressed in this article are solely those of the authors and do not necessarily represent those of their affiliated organizations, or those of the publisher, the editors and the reviewers. Any product that may be evaluated in this article, or claim that may be made by its manufacturer, is not guaranteed or endorsed by the publisher.
